# Hospital Meetings

**Published:** 1906-06-23

**Authors:** 


### HOSPITAL MEETINGS.

### ST. MARY'S HOSPITAL.

Mr. H. A. Harben, L.C.C., presided at a quarterly general meeting of the Governors on June 14, when the Board of Management submitted a report on the work of the hospital during the first five months of the year and on its financial position. The meeting was further made special, for the purpose of altering the laws of the hospital so as to enable the Board of Management to elect at any time to the honorary medical staff, under proper safeguards and conditions, two supernumerary members. This alteration is made in order to admit of the election to the staff of Dr. A. E. Wright, F.R.S., director in medical charge of the recently established department for therapeutic inoculation, an important new line of treatment which originated in St. Mary's Hospital through Dr. Wright.

It was the painful experience of the hospital staff to be compelled daily to deny admission to suitable patients because all the available beds were full. Yet coexistent with this experience is the fact that wards for sixty beds, in which 1,000 patients might be treated annually, lie empty in the Clarence wing for lack of the funds to furnish and maintain them. The district served by the hospital embraces Paddington, North Kensington, West Marylebone, Kilburn, Willesden, etc., and the Board specially comment on the fact that though Kensington and West Marylebone send their poor, they do not send their pence. The number of both in and out patients admitted to the hospital continues to grow, showing an increase of in-patients during the first five months of this year compared with the same period of 1905 (when the record number was reached) of 77, and of out-patients 678. The scope of relief afforded had also been extended by the establishment of the *x*-ray department, the therapeutic inoculation department, and the Dresden Assistance Fund, which, through the bequest of the late Mr. Edmund Dresden, has placed at the disposal of the hospital a sum of £865 annually for the benefit of in-patients on their discharge. The Board are thus enabled to send a greatly increased number of patients to convalescent homes and otherwise to help them. The Board would be grateful for special donations to be devoted to the organisation and development of the department for therapeutic inoculation, which work they feel is of high importance in the direction of rendering practicable the application of this method of treatment for the benefit of patients, and of facilitating further and more extended investigations for determining the scope of such therapeutic inoculations.

The Board report with much satisfaction that the decrease in last year's expenditure, when £12 per occupied bed was saved, continues this year, the expenditure during the first quarter of 1906 being, in fact, less than in the corresponding quarter of 1905. Unfortunately, however, the receipts for the first five months of this year fall far short of the expenditure, the former being £9,858, of which £8,550 is for general purposes, and the latter, representing £12,085, being at the rate of £2,417 per month. The deficiency in the present year on the maintenance of the hospital is no less than £3,535, a situation which is summarised by the Board in the words "increasing necessity for the hospital, increased work done, increased efficiency and economy, but decreased and extremely inadequate public support."

The report having been adopted, the retiring members of the Board and the four treasurers were re-elected, and Mr. Herbert Page, who for a great number of years has been on the staff of St. Mary's and is now retiring, was elected Vice-President.

### CENTRAL LONDON THROAT, NOSE, AND EAR HOSPITAL.

Captain Hutton, the Chairman, presided at the thirty-second annual meeting of the Central London Throat, Nose, and Ear Hospital, Gray's Inn Road, on June 13. The report presented stated that the in-patients numbered 354, as compared with 365 in 1904, the average residence being twelve days. Modern developments called for the adoption of operative procedure of a more serious kind, involving a longer residence of patients in the wards. The number admitted during the year had on that account been necessarily diminished, and there was an increased number waiting for vacant beds. The necessity for extended accommodation thus became all the more urgent, and the extension was being carried on with the utmost possible despatch. Of the 10,052 out-patients admitted 2,305 were sent in on the direct recommendation of medical practitioners, 6,219 gave a contribution to the funds, 3,017 were admitted free, and 816 presented a subscriber's letter. The number of patients on whom it had been necessary to perform various minor operations under anæsthetics had increased from 2,976 in 1903 to 3,780 in 1905. During the year 343 medical practitioners signed the visitors' book and witnessed the practice of the hospital. In addition there were 34 students, each giving regular attendance for three months and assisting in the preliminary examination of patients. In accordance with custom, lectures and demonstrations had been given during the winter months by members of the medical staff. The total ordinary income was £2,983, compared with £2,836 in the previous year. Of this, £1,807 was from patients' voluntary contributions, as against £1,914 in 1904. The expenditure was £2,670, as compared with £2,758 in 1904. It would be seen that the expenditure had been diminished, although there had been an increase in the relief afforded. The liabilities of the hospital at December 31, 1905, were £385, as against £1,380 in 1904—a very gratifying reduction. The Extension Fund had been augmented by donations of £774, and £6,000 was now in hand toward the new building. While the Committee would have desired to complete the entire scheme of enlargement, and so doubled the present accommodation, they hesitated to do more than complete a small portion, costing between £6,000 and £7,000. Realising the difficulties which had beset other institutions in not being able to defray the cost of erection of new buildings except out of borrowed money, nor in some cases of being able to meet the current expenses for future upkeep, the Committee were confident they had pursued the right policy in completing only the portion of the building for which they had funds in hand, and trusted that that policy would be an additional incentive to the benevolent to extend further assistance. During the past year Mr. Carmichael Thomas and Mr. Frederick Eiloart had rejoined the Committee of Management.

After service of nearly ten years, Miss Farmer had resigned her appointment as matron, and to the vacant office Miss Macdonald had been elected. Dr. Wyatt Wingrave had decided, with the approval of the Committee and of his colleagues, to confine himself to the pathological and educational branch of the hospital work. He had hitherto organised and conducted both departments with such ability and enthusiasm that an even greater success was anticipated in the future.

Captain Jessel moved the adoption of the report, which, he said, showed how admirably the work of the hospital had been carried on during the past year, and for this their warmest thanks were due to the Secretary and the Committee, who had so managed the funds at their disposal that not only had more relief been given, but it had cost less.

Mr. Stuart-Low seconded, and emphasised the need for greater accommodation.

The report was adopted, the retiring members of the Committee were re-elected, and votes of thanks to the medical officers, the nursing staff, the Secretary, Superintendent, and the Chairman closed the proceedings.

### WOMEN'S HOSPITAL, SOHO SQUARE.

The annual Court of Governors was held on June 14th, Mr. Victor Williamson, C.M.G., in the chair. The Chairman moved the adoption of the report, and remarked that in some ways it seemed to be a record one. They had had a larger number of in-patients than ever before, and at a lower cost. The medical report tended also to show what very good work the hospital was doing. Unfortunately they were not supported in the way they could wish. Their income fell short of their expenditure by £1,550. As long as he had been connected with the hospital they had always been about £2,000 short, and depended upon chance legacies —an unsatisfactory and fluctuating source of income. They had paid off £1,000 of their mortgage, but they still owed £6,000, and when they turned to their scheme for rebuilding he confessed he felt very disheartened. They had issued an appeal last November, and the lowest figure at which the estimates could be put was £40,000, with another £10,000 for furnishing, etc. Of that £50,000 they had got £5,000 promised in answer to their appeal. Their kind friends were constantly asking them when they were going to begin building, but the committee felt they could not embark on so large an expenditure with such very insufficient funds in hand, on the chance of people seeing the work in progress, and thereby being induced to subscribe. The committee were not, however, sitting still, but were considering various means of raising funds, among them a festival dinner next year, if a suitable President could be found. They owed a great debt of gratitude to Lady Samuel and the Ladies' Association; the enthusiasm with which they had taken up the interests of the hospital could not be exaggerated, and he desired to tender them the best thanks of the committee and of the governors. The motion was seconded by Captain G. A. Webbe, and carried, as were various other resolutions, and the proceedings then terminated.

